#  Telehealth Expansion, Internet Speed, and Primary Care Access Before and During COVID-19

**DOI:** 10.1001/jamanetworkopen.2023.47686

**Published:** 2024-01-05

**Authors:** Alyssa Shell Tilhou, Arjun Jain, Thomas DeLeire

**Affiliations:** 1 Boston University Medical Center, Boston, Massachusetts; 2 University of Wisconsin-Madison; 3 Georgetown University, Washington, DC

## Abstract

**Importance:**

Primary care (PC) receipt is associated with better health outcomes. How telehealth expansion and internet speed are associated with PC use is unclear.

**Objective:**

To examine the association of telehealth and internet speed with PC use across sociodemographic determinants of health.

**Design, Setting, and Participants:**

This cohort study performed difference-in-differences regression of the change in in-person and telehealth PC visits between pre–COVID-19 public health emergency (PHE) (June 1, 2019, to February 29, 2020) and an initial (March 1, 2020, to May 31, 2020) and prolonged (March 1, 2020, to December 31, 2021) PHE period among continuously enrolled nonpregnant, nondisabled Wisconsin Medicaid beneficiaries aged 18 to 64 years. Data were analyzed from March 2022 to March 2023.

**Exposure:**

PHE-induced telehealth expansion.

**Main Outcomes and Measures:**

Change in PC telehealth (using *Current Procedural Terminology *codes) visits: (1) count; (2) visit share completed by telehealth; (3) percentage of PHE-induced visit decline offset by telehealth. High-speed internet (HSI) defined as living in a census block group with a median block maximum download speed of 940 megabits per second or greater (June 2020 Federal Communications Commission broadband data); other census block groups classified as low-speed internet (LSI).

**Results:**

In the total cohort of 172 387 participants, 102 989 (59.7%) were female, 103 848 (60.2%) were non-Hispanic White, 34 258 (19.9%) were non-Hispanic Black, 15 020 (8.7%) were Hispanic, 104 239 (60.5%) were aged 26 to 45 years, and 112 355 (66.0%) lived in urban counties. A total of 142 433 (82.6%) had access to HSI; 72 524 (42.1%) had a chronic condition. There was a mean (SD) of 0.138 (0.261) pre-PHE PC visits per month. In the pre-PHE period, visit rates were significantly higher for female than male participants, non-Hispanic White than non-Hispanic Black individuals, urban than rural residents, those with HSI than LSI, and patients with chronic disease than patients without. In the initial PHE period, female participants had a greater increase in telehealth visits than male participants (43.1%; 95% CI, 37.02%-49.18%; *P* < .001), share (2.20 percentage point difference [PPD]; 95% CI, 1.06-3.33 PPD; *P* < .001) and offset (6.81 PPD; 95% CI, 3.74-9.87 PPD; *P* < .001). Non-Hispanic Black participants had a greater increase in share than non-Hispanic White participants (5.44 PPD; 95% CI, 4.07-6.81 PPD; *P* < .001) and offset (15.22 PPD; 95% CI, 10.69-19.75 PPD; *P* < .001). Hispanic participants had a greater increase in telehealth visits than Non-Hispanic White participants (35.60%; 95% CI, 25.55%-45.64%; *P* < .001), share (8.50 PPD; 95% CI, 6.75-10.26 PPD; *P* < .001) and offset (12.93 PPD; 95% CI, 6.25-19.60 PPD; *P* < .001). Urban participants had a greater increase in telehealth visits than rural participants (63.87%; 95% CI, 52.62%-75.11%; *P* < .001), share (9.13 PPD; 95% CI, 7.84-10.42 PPD; *P* < .001), and offset (13.31 PPD; 95% CI; 9.62-16.99 PPD; *P* < .001). Participants with HSI had a greater increase in telehealth visits than those with LSI (55.23%; 95% CI, 42.26%-68.20%; *P* < .001), share (6.61 PPD; 95% CI, 5.00-8.23 PPD; *P* < .001), and offset (6.82 PPD; 95% CI, 2.15-11.49 PPD; *P* = .004). Participants with chronic disease had a greater increase in telehealth visits than those with none (188.07%; 95% CI, 175.27%-200.86%; *P* < .001), share (4.50 PPD; 95% CI, 3.58-5.42 PPD; *P* < .001), and offset (9.03 PPD; 95% CI, 6.01-12.04 PPD; *P* < .001). Prolonged PHE differences were similar. Differences persisted among those with HSI.

**Conclusions and Relevance:**

In this cohort study of Wisconsin Medicaid beneficiaries, greater telehealth uptake occurred in groups with higher pre-PHE utilization, except for high uptake among Hispanic and non-Hispanic Black individuals despite low pre-PHE utilization. HSI did not moderate disparities. These findings suggest telehealth and HSI may boost PC receipt, but will generally not close utilization gaps.

## Key Points

**Question:**

What disparities emerged in access to primary care telehealth services during the COVID-19 pandemic, and to what degree is access to high-speed internet associated with disparities in telehealth utilization?

**Findings:**

In this cohort study of 172 387 Wisconsin Medicaid beneficiaries, telehealth expansion exposed disparities in utilization of telehealth services that persisted even among beneficiaries with high-speed internet.

**Meaning:**

These findings suggest that expansion of telehealth service or access to high-speed internet is unlikely to close gaps in utilization of primary care services.

## Teaser

This cohort study evaluates the association of telehealth and internet speed with primary care use across sociodemographic determinants of health.

## Introduction

Telehealth has long been identified as a strategy to expand health care access while reducing health care costs.^1,2,3,4^ Yet, for decades telehealth represented a minority of health care services.^4,5^ The COVID-19 pandemic dramatically elevated telehealth’s contribution as health systems shifted toward remote care delivery to minimize risk for patients and staff.^6^ Reliance on telehealth has eased with the pandemic’s denouement, but telehealth remains a prominent modality of health care engagement.^7,8^

As telehealth solidifies its position in the health care landscape, telehealth utilization has become increasingly relevant for health equity^9^ because telehealth both mitigates and exposes barriers to care. For example, telehealth minimizes barriers such as geography and transportation by delivering services remotely.^10,11^ Telehealth also enhances scheduling flexibility and decreases logistical challenges. Telehealth could thus close a variety of utilization gaps associated with socioeconomic status (SES), race, rurality, and age.^12,13,14,15^

However, telehealth requires equipment, internet, and digital literacy. Disparities in ability to access and use digital technologies due to factors like age, race, SES, education, and geography constitute a social phenomenon known as the digital divide.^16,17^ In the context of telehealth expansion, a digital divide could contribute to inequity in health care utilization.^18^ Early studies of telehealth uptake during the pandemic demonstrated disparities in telehealth utilization and modality (audio vs audio and video) by race, ethnicity, and income.^8,19,20,21,22,23,24,25^ Notably, this COVID-19–manifest digital divide rests upon a foundation of unequal access to health care shaped by parallel socioeconomic, environmental, and political determinants of health.^26,27,28,29,30^ Consequently, telehealth expansion could unwittingly deepen preexisting disparities, raising the likelihood of disproportionate morbidity and premature mortality for those already at greater risk.^28,31^

Of the various factors impacting telehealth utilization, high-speed internet (HSI) may play a pivotal role in promoting telehealth.^8,19,20,32^ In rural^33^ and urban areas alike,^34^ access to broadband internet is associated with greater individual- and neighborhood-level telehealth use.^20,35^ As such, broadband availability may represent a key policy intervention to promote telehealth equity. Over one-quarter of the US population lacks broadband in their homes, concentrated among individuals with annual incomes less than $30 000.^36,37^ However, the degree to which internet access moderates the effect of social determinants on telehealth utilization remains unclear. Moreover, while literature has examined telehealth expansion within the Medicaid program,^4,38^ little research has described the association between broadband access and telehealth utilization within Medicaid.

Although structural vulnerabilities are associated with access to care, medical diagnoses are primary motivators of health care engagement. Individuals with poor health also face serious consequences after missing needed care. As such, analyses examining utilization trends can be strengthened by evaluating variation by health status.

This article leverages a period of rapid telehealth expansion in the Wisconsin Medicaid program during the COVID-19 public health emergency (PHE) to comprehensively evaluate the association between social determinants of health and telehealth utilization. Specifically, we asked the following questions: (1) whether, during the PHE, telehealth uptake differed by sociodemographic characteristics such as race, sex, age, education, income, and geography; (2) whether telehealth use increased to a greater extent among beneficiaries residing in areas with HIS; (3) whether access to HSI moderated differences in telehealth utilization by beneficiary characteristics; and (4) whether associations between access to HSI, beneficiary characteristics, and telehealth use varied by health status. By answering these questions, we sought to delineate the association between telehealth expansion, HSI, and access to primary care for vulnerable populations in Wisconsin.

## Methods

This study was determined exempt from review and informed consent by the University of Wisconsin institutional review board (common rule, category 5). This study followed Strengthening the Reporting of Observational Studies in Epidemiology (STROBE) reporting guidelines. The data for this study came from Wisconsin Medicaid enrollment and claims December 1, 2018, to December 31, 2021. Enrollment data identified enrollment continuity and sociodemographic characteristics. Claims identified primary care setting, visit modality, and diagnosis codes. Federal Communications Commission (FCC) Fixed Broadband Deployment Data from June 2020 supplied internet speed. We used the FCC-maintained list of Wisconsin census blocks to map HSI availability to beneficiaries. The study cohort included nonpregnant, nondisabled adults aged 18 to 64 years eligible for Wisconsin Medicaid as parents or caretakers or childless adults. We required continuous enrollment during the study period (June 1, 2019, to December 31, 2021).

### Outcomes and Covariates

The primary outcomes were monthly telehealth and in-person primary care visits. We defined primary care visits using a combination of practitioner specialty and facility codes. To distinguish telehealth from in-person visits, we used procedure codes, place of service codes, or modifiers indicating telehealth (eMethods 1 and eMethods 2 in Supplement 1). We defined 3 time periods: pre-PHE (June 1, 2019, to February 29, 2020), initial PHE (March 1, 2020, to May 31, 2020), and prolonged PHE (March 1, 2020, to December 31, 2021).

To understand utilization differences by health status, we defined 3 nonmutually exclusive subgroups defined by having chronic medical disease (CMD), chronic psychiatric disease (CPD), and substance use disorder (SUD). Subgroup assignment required 1 or more claim with a relevant *International Statistical Classification of Diseases and Related Health Problems, Tenth Revision (ICD-10)* diagnosis in the outpatient, inpatient, or emergency department setting in the 6-month period before study start (December 1, 2018 to May 31, 2019). CMD diagnoses included asthma, chronic kidney disease, chronic obstructive pulmonary disease, coronary artery disease, diabetes, hypertension, heart failure, thyroid disorders, and osteoarthritis. CPD diagnoses included all *ICD-10* F codes except for SUDs or acute conditions. SUD diagnoses included alcohol, opioid, cannabis, sedative, stimulant, and other psychoactive SUDs excluding nicotine and miscellaneous SUDs. See eMethods 3 in Supplement 1 for more details.

We evaluated the following sociodemographic characteristics: age, sex, race, ethnicity, income (percentage of the federal poverty level [FPL]), education, geography (rural or urban county residence), and access to HSI. In these data, race and ethnicity are obtained by self-identification but occasionally reported by caseworkers. Race was assessed in this study in order to examine whether race is associated with telehealth utilization. To determine HSI access, cohort members were assigned to a census block group using their 9-digit zip code of residence in March 2020. Census block groups were matched to FCC data. HSI (vs low-speed internet [LSI]) was defined as living in a census block group where the median block had a maximum available download speed of 940 megabits per second or higher according to the distribution of internet speed in our data and documented telehealth requirements for download and upload speed.^39^

### Statistical Analysis

#### Differences in Use of Telehealth by Beneficiary Characteristic

Mean pre-PHE visit rates were estimated for each sociodemographic characteristic, and differences in means were tested using *t* tests (eg, female participants vs male participants). Difference-in-difference estimates from linear regression measured post-PHE changes in telehealth visits, in-person visits, and the share of total visits completed by telehealth (hereafter, telehealth share). For each factor, we estimated 2 models to evaluate the change in visits between pre-PHE and the initial and prolonged PHE periods. Models controlled for sociodemographic characteristics, presence of chronic disease (in any of the 3 categories), access to HSI, and a term interacting PHE period and the factor of interest. Regression coefficients were used to calculate 3 measures for each characteristic (eg, male participants vs female participants): (1) the percentage difference in telehealth visit increase, (2) the percentage point difference (PPD) in telehealth share, and (3) the PPD in pre-PHE visit decline offset by the increase in telehealth (hereafter, telehealth offset). Standard errors were calculated using the delta method, with clustering at the census block group level.

#### Differences in Use of Telehealth by Access to HSI

Additional difference-in-difference models were estimated to measure post-PHE changes in telehealth visits, in-person visits, and telehealth share for beneficiaries with and without access to HSI. Models controlled for sociodemographic characteristics, presence of chronic disease, and a term interacting PHE period and HSI. Model coefficients were again used to calculate the 3 measures of differences in telehealth utilization by access to HSI.

#### Stratifying by Access to High-Speed Internet

To assess if HSI access moderated telehealth use by beneficiary characteristic, we reestimated models stratifying by access to HSI. Differences in telehealth utilization by beneficiary characteristic among only beneficiaries with or without access to HSI would suggest moderation.

#### Variation in Differences in Use of Telehealth by Health Status

To assess whether associations between HSI, beneficiary characteristics, and telehealth use vary by health status, we repeated analyses for CMD, CPD, and SUD subgroups. As above, difference-in-difference models estimated differences in telehealth visits, in-person visits, and telehealth share, and coefficients were used to calculate telehealth utilization measures.

All analyses were conducted using Stata statistical software version 17.0 (StataCorp) between March 2022 and March 2023. All hypothesis tests were 2-sided. The statistical significance level was set at .05. Parallel trends were examined and no meaningful differences identified.

## Results

In the cohort of 172 387 participants, 102 989 (59.7%) were female, 103 848 (60.2%) were non-Hispanic White, 34 258 (19.9%) were non-Hispanic Black, 15 020 (8.7%) were Hispanic, and 104 239 (60.5%) were aged 26 to 45 (Table 1). Most individuals reported urban residence (112 355 participants [66.0%]), completing high school or more (100 494 participants [59.4%]), and income 50% or less of the FPL (109 796 participants [61.1%]). A total of 142 433 (82.6%) lived with access to HSI and 72 524 (42.1%) had a chronic condition. There was a mean (SD) of 0.138 (0.261) primary care visits per month pre-PHE.

**Table 1.** Sociodemographic and Clinical Characteristics of Sample

Characteristic	No. (%)	Pre-PHE visits per month^a^	*P* value^b^	Difference (95% CI)
Sex				
Female	102 989 (59.7)	0.150	<.001	0.030 (0.028 to 0.033)
Male	69 398 (40.3)	0.120	NA	[Reference]
Race and Ethnicity				
Black non-Hispanic	34 258 (19.9)	0.111	<.001	−0.037 (−0.040 to −0.033)
Hispanic	15 020 (8.7)	0.143	0.07	−0.004 (−0.009 to 0.000)
Other Race non-Hispanic^c^	12 938 (7.5)	0.130	<.001	−0.017 (−0.022 to −0.012)
White non-Hispanic	103 848 (60.2)	0.148	NA	[Reference]
Missing race or ethnicity	6323 (3.7)	0.126	<0.001	−0.021 (−0.028 to −0.014)
Income				
≤50% FPL	109 796 (61.1)	0.141	NA	[Reference]
>50%-100% FPL	52 119 (27.3)	0.133	<.001	−0.007 (−0.011 to −0.005)
>100% FPL	10 466 (11.5)	0.131	<.001	−0.010 (−0.016 to −0.005)
Missing income	<10 (0.0)	0.074	0.56	−0.067 (−0.293 to −0.159)
County				
Rural	38 919 (23.0)	0.123	NA	[Reference]
Urban	112 355 (66.0)	0.143	<.001	0.020 (0.017 to 0.023)
Missing	21 113 (11.0)	0.136	<0.001	0.013 (0.009 to 0.017)
Internet access				
Low-speed	23 400 (13.6)	0.114	NA	[Reference]
High-speed	142 433 (82.6)	0.142	<.001	0.028 (0.025 to 0.032)
Missing	6554 (3.8)	0.128	<0.001	0.014 (0.008 to 0.021)
Education				
Less than high school	30 257 (17.0)	0.129	NA	[Reference]
High school or more	100 494 (59.4)	0.144	<.001	0.015 (0.012 to 0.018)
Missing	41 636 (23.6)	0.131	0.25	0.002 (−0.002 to 0.006)
Age, y				
18-25	22 687 (13.2)	0.096	<.001	−0.034 (−0.038 to −0.030)
26-35	56 226 (32.6)	0.130	NA	[Reference]
36-45	48 013 (27.9)	0.150	<.001	0.020 (0.017 to 0.023)
46-55	30 424 (17.6)	0.159	<.001	0.029 (0.025 to 0.033)
56-64	15 037 (8.7)	0.148	<.001	0.018 (0.014 to 0.023)
Comorbidities				
Any chronic condition	72 524 (42.1)	0.211	<.001	0.126 (0.124 to 0.129)
No chronic condition	99 863 (57.9)	0.085	NA	[Reference]
Chronic medical condition	37 671 (21.9)	0.223	NA	[Reference]
Chronic psychiatric condition	47 017 (27.3)	0.219	NA	[Reference]
Substance use disorder	16 837 (9.8)	0.278	NA	[Reference]
Unique persons	172 387	NA	NA	[Reference]
Person-months	5 343 997	NA	NA	NA

Abbreviations: FPL, federal poverty level; NA, not applicable; PHE, public health emergency.

^a^ Estimated pre-PHE telehealth visit rates were 0.001 or less per month for all characteristics.

^b^ *P* value for test of difference between pre-PHE visit rates for each characteristic.

^c^ Other race non-Hispanic includes American Indian, Asian, and Pacific Islander.

The Figure shows mean monthly primary care visits by modality. At PHE onset, total and in-person visits plummeted alongside a sudden steep rise in telehealth. Telehealth visits peaked in April 2020, representing more than 50% of visits. This percentage slowly declined and stabilized near 8% in late 2021. Total visits remained below pre-PHE levels through 2021.

**Figure.**
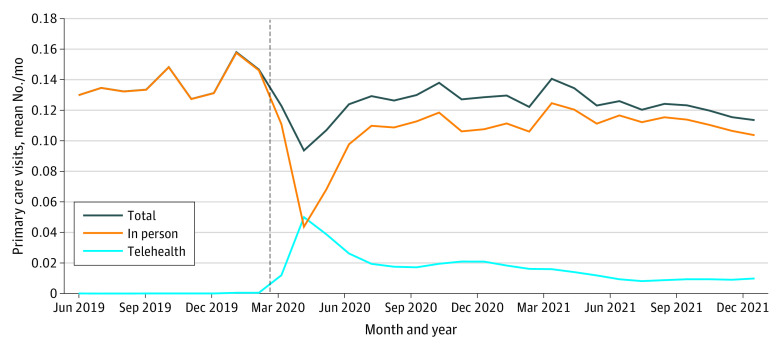
Primary Care Visits per Month Vertical line indicates the start of the public health emergency, designated in this study as March 1, 2020.

### Differences in Telehealth Utilization by Beneficiary Characteristic

#### Geography

Pre-PHE, visits were higher among urban than rural residents (difference, 0.020 visits; 95% CI, 0.017-0.023 visits; *P* < .001). During the PHE, telehealth use increased more among urban than rural residents, widening the pre-PHE visit gap (Table 2). Post-PHE, urban residents exhibited a 63.87% greater increase in visits (initial: 95% CI, 52.62%-75.11%; *P* < .001; prolonged: 95% CI, 53.42%-72.72%; *P* < .001) and a greater increase in telehealth share (initial: 9.13 PPD; 95% CI, 7.84-10.42 PPD; *P* < .001; prolonged: 3.94 PPD; 95% CI, 3.35-4.52 PPD; *P* < .001) and telehealth offset (13.31 PPD; 95% CI, 9.62-16.99 PPD; *P* < .001).

**Table 2.** Increases in the Use of Telehealth (TH) by Access to High-Speed Internet and by Demographic Characteristic^a^

Characteristic	Change in monthly TH visits		Change in TH share		TH offset
	June 2019 to May 2020	June 2019 to December 2021	June 2019 to May 2020	June 2019 to December 2021	June 2019 to May 2020
County					
Rural	0.02	0.01	24.56	11.11	42.02
Urban	0.04	0.02	33.69	15.05	55.33
Difference (SE) [95% CI]	63.87% (5.74%) [52.62% to 75.11%]^b^	63.07% (4.92%) [53.42% to 72.72%]^b^	9.13 (0.66) [7.84 to 10.42] PPD^b^	3.94 (0.30) [3.35 to 4.52] PPD^b^	13.31 (1.88) [9.62 to 16.99] PPD^b^
Sex					
Female	0.04	0.02	32.65	14.93	55.18
Male	0.03	0.01	30.46	12.80	48.37
Difference (SE) [95% CI]	43.10% (3.10%) [37.02% to 49.18%]^b^	56.37% (3.02%) [50.44% to 62.30%]^b^	2.20 (0.58) [1.06 to 3.33] PPD^b^	2.12 (0.28) [1.58 to 2.67 PPD]^b^	6.81 (1.56) [3.74 to 9.87] PPD^b^
Race and Ethnicity					
Black non-Hispanic	0.03	0.02	35.47	14.78	64.13
Hispanic	0.04	0.02	38.53	18.13	61.84
Other non-Hispanic^c^	0.03	0.01	31.74	11.73	53.32
White non-Hispanic	0.03	0.02	30.03	13.84	48.91
Black vs White difference (SE) [95% CI]	1.52% (4.29%) [−6.88% to 9.93%]	−10.19% (3.66%) [−17.36% to −3.00%]^d^	5.44 (0.70) [4.07 to 6.81] PPD^b^	0.94 (0.35) [0.26 to 1.62] PPD^d^	15.22 (2.31) [10.69 to 19.75] PPD^b^
Hispanic vs White difference (SE) [95% CI]	35.60% (5.12%) [25.55% to 45.64%]^b^	37.72% (4.42%) [29.06% to 46.38%]^b^	8.50 (0.90) [6.75 to 10.26] PPD^b^	4.29 (0.46) [3.38 to 5.19] PPD^b^	12.93 (3.41) [6.25 to 19.60] PPD^b^
Other vs White difference (SE) [95% CI]	−5.71% (4.95%) [−15.42% to 3.99%]	−23.56% (3.46%) [−30.34% to −16.79%]^b^	1.71 (1.21) [−0.66 to 4.07] PPD	−2.11 (0.46) [−3.02 to −1.20] PPD^b^	4.41 (3.73) [−2.90 to 11.72] PPD
Income					
≤50% FPL	0.03	0.02	32.35	14.80	53.02
>50%-100% FPL	0.03	0.02	31.14	13.23	51.35
>100% FPL	0.03	0.01	31.52	13.32	55.71
Moderate to low difference (SE) [95% CI]	−4.21% (2.30%) [−8.71% to 0.29%]	−8.03% (1.86%) [−11.67% to −4.40%]^b^	−1.21 (0.56) [−2.31 to −0.12] PPD	−1.58 (0.23) [−2.03 to −1.12] PPD^b^	−1.67 (1.75) [−5.10 to 1.76] PPD
High to low difference (SE) [95% CI]	−9.45% (3.13%) [−15.58% to −3.32%]^d^	−18.85% (2.21%) [−23.19% to −14.52%]^b^	−0.82 (0.75) [−2.30 to 0.65] PPD	−1.49 (0.29) [−2.06 to −0.91] PPD^b^	2.69 (2.57) [−2.35 to 7.74] PPD
Education					
Less than high school	0.04	0.02	35.09	15.38	42.83
High school or more	0.03	0.02	31.41	14.25	53.71
Difference (SE) [95% CI]	−4.8% (3.20%) [−11.07% to 1.47%]	2.16% (2.93%) [−3.57% to 7.90%]	−3.68 (0.71) [−5.06 to −2.29] PPD^b^	−1.13 (0.36) [−1.85 to −0.42] PPD^d^	−11.83 (3.16) [−18.02 to −5.63] PPD^b^
Age, y					
18-25	0.02	0.01	28.17	13.17	49.17
26-35	0.03	0.02	32.15	15.61	53.72
36-45	0.04	0.02	32.68	14.94	57.17
46-55	0.04	0.02	32.80	13.11	52.55
56-64	0.03	0.01	30.36	10.92	41.45
18-25 vs 26-35 difference (SE) [95% CI]	−34.54% (2.63%) [−39.69% to −29.38%]^b^	−33.71% (2.13%) [−37.89% to −29.52%]^b^	−3.98 (0.84) [−5.63 to −2.32] PPD^b^	−2.43 (0.34) [−3.10 to −1.76] PPD^b^	−4.55 (2.8) [−10.04 to 0.94] PPD
36-45 vs 26-35 difference (SE) [95% CI]	19.61% (3.07%) [13.60% to 25.62%]^b^	12.33% (2.55%) [7.32% to 17.33%]^b^	0.53 (0.57) [−0.59 to 1.66] PPD	−0.67 (0.26) [−1.18 to −0.15] PPD^e^	3.45 (2.13) [−0.72 to 7.62] PPD
46-55 vs 26-35 difference (SE) [95% CI]	23.36% (3.60%) [16.29% to 30.42%]^b^	5.94% (2.87%) [0.31% to 11.57%]^e^	0.65 (0.66) [−0.64 to 1.93] PPD	−2.49 (0.29) [−3.06 to −1.93] PPD^b^	−1.17 (2.33) [−5.74 to 3.40] PPD
56-64 vs 26-35 difference (SE) [95% CI]	−0.79% (3.88%) [−8.40%-6.82%]	−16.81% (2.94%) [−22.58% to −11.04%]^b^	−1.79 (0.86) [−3.48 to −0.09] PPD^e^	−4.68 (0.34) [−5.35 to −4.02] PPD^b^	−12.27 (2.26) [−16.70 to −7.83] PPD^b^
Comorbidities					
Any chronic	0.05	0.03	33.56	15.57	56.08
No chronic	0.02	0.01	29.06	11.96	47.06
Difference (SE) [95% CI]	188.07% (6.53%) [175.27% to 200.86%]^b^	185.41% (5.76%) [174.08% to 196.65%]^b^	4.50 (0.47) [3.58 to 5.42] PPD^b^	3.61 (0.19) [3.24 to 3.99] PPD^b^	9.03 (1.54) [6.01 to 12.04] PPD^b^
Internet					
High speed	0.04	0.02	32.70	14.47	53.55
Low speed	0.02	0.01	26.09	12.23	46.73
Difference (SE) [95% CI]	55.23% (6.62%) [42.26% to 68.20%]^b^	50.22% (5.62%) [39.21% to 61.23%]^b^	6.61 (0.82) [5.00 to 8.23] PPD^b^	2.23 (0.41) [1.43 to 3.04 PPD]^b^	6.82 (2.38) [2.15 to 11.49] PPD^d^
No. (person-months)	2 068 644	5 343 997	222 112	558 348	2 068 644

Abbreviations: FPL, federal poverty limit; PPD, percentage point difference.

^a^ Estimates of the change in telehealth visits per month, the change in the share of visits that were telehealth, and the change in in-person visits are from a set of difference-in-difference models where each characteristic is interacted with a post-PHE indicator while controlling for all other characteristics and clustering at the census block group level. Reported differences reflect either the difference in percentage change or difference in percentage points. Standard errors on the differences are calculated by the delta method.

^b^ *P* < .001.

^c^ Other race non-Hispanic includes American Indian, Asian, and Pacific Islander.

^d^ *P* < .01.

^e^ *P* < .05.

#### Sex

Pre-PHE, visits were higher among female than male beneficiaries (difference, 0.030 visits; 95% CI, 0.028-0.033 visits; *P* < .001). Post-PHE, they also experienced greater telehealth uptake (Table 2). Relative to male participants, female participants exhibited a 43.1% (95% CI, 37.02%-49.18%; *P* < .001) and 56.37% (95% CI, 50.44%-62.30%; *P* < .001) greater visit increase in the initial and prolonged PHE, respectively. Female participants also exhibited a greater increase in telehealth share (initial: 2.20 PPD; 95% CI, 1.06-3.33 PPD; *P* < .001; prolonged: 2.12 PPD; 95% CI, 1.58-2.67 PPD; *P* < .001) and offset (6.81 PPD; 95% CI, 3.74-9.87 PPD; *P* < .001).

#### Race and Ethnicity

Pre-PHE, relative to non-Hispanic White individuals, non-Hispanic Black individuals exhibited significantly lower visit rates (difference, −0.037 visits; 95% CI, −0.040 to −0.033 visits; *P* < .001). In contrast, there was no difference in pre-PHE visit rates for Hispanic relative to non-Hispanic White individuals. In the prolonged PHE, telehealth visits increased 10.19% less for non-Hispanic Black individuals than for non-Hispanic White individuals (95% CI, −17.37% to −3.00%; *P* = .005), further widening the pre-PHE gap (Table 2). However, non-Hispanic Black individuals exhibited a greater increase in telehealth share (initial: 5.44 PPD; 95% CI, 4.07 to 6.81 PPD; *P* < .001; prolonged: 0.94 PPD; 95% CI, 0.26 to 1.62 PPD; *P* = .007) and telehealth offset (15.22 PPD; 95% CI, 10.69 to 19.75 PPD; *P* < .001) than non-Hispanic White individuals. Consistently, Hispanic individuals exhibited greater telehealth uptake than non-Hispanic White individuals. In the initial PHE, Hispanic individuals exhibited a 35.60% greater increase in telehealth visits (95% CI, 22.55% to 45.64%; *P* < .001), share (8.50 PPD; 95% CI, 6.75 to 10.26 PPD; *P* < .001), and offset (12.93 PPD; 95% CI, 6.25 to 19.60 PPD; *P* < .001). This advantage persisted in the prolonged PHE. A consistent pattern did not emerge for individuals of other non-Hispanic races.

#### Income

Pre-PHE, the highest income group exhibited a lower visit rate than the lowest income group (difference, −0.010 visits; 95% CI, −0.016 to −0.005 visits; *P* < .001). During the initial and prolonged PHE, individuals in the lowest income group exhibited greater telehealth uptake than those in higher income groups, widening the preexisting visit gap (Table 2). Relative to the lowest income group, the moderate income group exhibited an 8.03% smaller increase in the prolonged PHE (95% CI, −11.67% to −4.40%; *P* < .001) while the highest income group exhibited a 9.45% smaller increase the initial PHE (95% CI, −15.58% to −3.32%; *P* = .003) and a 18.85% smaller increase in in the prolonged PHE (95% CI, −23.19% to −14.52%; *P* < .001). Generally, the moderate and highest income groups also exhibited a smaller increase in telehealth share relative to the lowest income group. There were no differences observed in telehealth offset by income group.

#### Education

Pre-PHE, visit rates were higher among individuals with more education (difference, 0.015 visits; 95% CI, 0.012 to 0.018 visits; *P* < .001). Post-PHE, there were no significant differences in telehealth visit counts (Table 2). The higher education group exhibited a smaller increase in telehealth share (initial PHE: −3.68 PPD; 95% CI, −5.06 to −2.29 PPD; *P* < .001; prolonged PHE: −1.13 PPD; 95% CI, −1.85 to −0.42 PPD; *P* = .002) and offset (−11.83 PPD; 95% CI, −18.02 to −5.63 PPD; *P* < .001) than the lower education group.

#### Age

Pre-PHE, relative to those aged 26 to 35 years, visit rates were lower among those aged 18 to 25 years (difference, −0.034 visits; 95% CI −0.038 to −0.030 visits; *P* < .001). During the initial and prolonged PHE, telehealth use increased less among the youngest and oldest groups relative to those aged 26 to 35 years (Table 2). The youngest group experienced a one-third smaller increase in telehealth visits in the initial (−34.54 PPD; 95% CI, −39.69 to −29.38 PPD; *P* < .001) and prolonged PHE (−33.71 PPD; 95% CI, −37.89 to −29.52 PPD; *P* < .001). The oldest group exhibited a smaller increase in telehealth visits in the prolonged PHE (−16.81%; 95% CI, −22.58% to −11.04%; *P* < .001). In both the initial and prolonged PHE, the telehealth share increased less among the youngest (initial PHE: −3.98 PPD; 95% CI, −5.63 to −2.32 PPD; *P* < .001; prolonged PHE: −2.43 PPD; 95% CI, −3.10 to −1.76 PPD; *P* < .001) and oldest (initial PHE: −1.79 PPD; 95% CI, −3.48 to −0.09 PPD; *P* = .04; prolonged PHE: −4.68 PPD; 95% CI, −5.35 to −4.02 PPD; *P* < .001) groups relative to those aged 26 to 35 years. The oldest group exhibited a smaller telehealth offset than those aged 26 to 35 years (−12.27 PPD; 95% CI, −16.70 to −7.83 PPD; *P* < .001).

#### Chronic Disease

Pre-PHE, individuals with chronic disease completed more primary care visits than those without (difference, 0.126 visits; 95% CI, 0.124-0.129 visits; *P* < .001). Post-PHE, those with chronic disease exhibited a 188.07% greater telehealth visit increase in the initial PHE than those without (95% CI, 175.27%-200.86%; *P* < .001), which persisted in the prolonged PHE (95% CI, 174.08%-196.65%; *P* < .001) (Table 2). They also exhibited a greater increase in telehealth share (initial: 4.50 PPD; 95% CI, 3.58-5.42 PPD; *P* < .001; prolonged: 3.61 PPD; 95% CI, 3.24-3.99 PPD; *P* < .001) and offset (9.03 PPD; 95% CI, 6.01-12.04 PPD; *P* < .001).

### Differences in Telehealth Utilization by Access to HSI

Pre-PHE, individuals with HSI exhibited greater primary care utilization than those without (difference, 0.028 visits; 95% CI, 0.025-0.032 visits; *P* < .001). They also exhibited greater telehealth uptake by visit counts (initial PHE: 55.23%; 95% CI, 42.26%-68.20%; *P* < .001; prolonged PHE, 50.22%; 95% CI, 39.21%-61.23%; *P* < .001), telehealth share (initial PHE: 6.61 PPD; 95% CI, 5.00-8.23 PPD; *P* < .001; prolonged PHE: 2.23 PPD; 95% CI, 1.43-3.04 PPD; *P* < .001) and telehealth offset (6.82 PPD; 95% CI, 2.15-11.49 PPD; *P* = .004) (Table 2).

### Stratifying by Access to HSI

We reestimated models stratifying by internet speed (Table 3). Many differences persisted in both HSI and LSI areas including by geography, sex, age, and chronic disease. HSI exaggerated the telehealth advantage for females and urban residents in the initial PHE. However, stratification isolated the telehealth advantage for non-Hispanic Black and Hispanic individuals to those with HSI. In addition, individuals with more education (who completed higher visits pre-PHE) exhibited a smaller increase in telehealth share and offset relative to individuals with less education only among those with HSI.

**Table 3.** Increases in the Use of Telehealth (TH) by Demographic Characteristic and Access to High-Speed Internet^a^

Characteristic	Change in TH visits				Change in TH share				TH offset	
	High speed		Low speed		High speed		Low speed		High speed	Low speed
	Initital PHE	Prolonged PHE	June 2019 to May 2020	June 2019 to December 2021	June 2019 to May 2020	June 2019 to December 2021	June 2019 to May 2020	June 2019 to December 2021	June 2019 to May 2020	June 2019 to May 2020
County										
Rural	0.02	0.01	0.02	0.01	24.87	11.24	24.00	10.88	40.89	44.22
Urban	0.04	0.02	0.03	0.02	33.88	15.04	30.00	15.23	55.56	50.31
Difference (SE) [95% CI]	57.11% (6.47%) [44.42% to 69.79%]^b^	52.72% (5.43%) [42.09% to 63.36%]^b^	35.00% (10.90%) [13.65% to 56.36%]^c^	56.23% (10.21%) [36.24% to 76.28%]^b^	9.01 (0.80) [7.45 to 10.57] PPD^b^	3.79 (0.34) [3.13 to 4.46] PPD^b^	6.00 (1.68) [2.72 to 9.29] PPD^b^	4.34 (0.74) [2.89 to 5.80] PPD^b^	14.67 (2.16) [10.43 to 18.91] PPD^b^	6.10 (4.64) [−3.00 to 15.19] PPD
Sex										
Female	0.04	0.02	0.03	0.01	33.41	15.17	26.68	12.95	55.79	49.67
Male	0.03	0.01	0.02	0.01	31.27	13.06	24.95	11.07	49.29	41.58
Difference (SE) [95% CI]	42.83% (3.32%) [36.32% to 49.33%]^b^	56.46% (3.24%) [50.11% to 62.81%]^b^	38.58% (8.81%) [21.30% to 55.85%]^b^	49.12% (8.35%) [32.77% to 65.48%]^b^	2.14 (0.62) [0.92 to 3.36] PPD^c^	2.11 (0.30) [1.52 to 2.71] PPD^b^	1.73 (1.24) [−0.69 to 4.15] PPD	1.88 (0.57) [0.76 to 3.00] PPD^c^	6.50 (1.70) [3.17 to 9.83] PPD^b^	8.09 (3.76) [0.71 to 15.46] PPD^d^
Race and Ethnicity										
Black non-Hispanic	0.03	0.02	0.05	0.02	35.47	14.77	35.08	15.90	63.90	202.17
Hispanic	0.05	0.02	0.03	0.01	38.80	18.33	29.24	11.24	61.68	67.87
Other non-Hispanic^e^	0.03	0.01	0.03	0.01	33.42	12.17	20.61	8.87	54.69	43.87
White non-Hispanic	0.04	0.02	0.02	0.01	30.73	14.08	26.41	12.58	49.34	46.35
Black vs White difference (SE) [95% CI]	−6.25% (4.00%) [−14.09% to 1.58%]	−16.92% (3.42%) [−23.62% to −10.22%]^b^	100.84% (66.09%) [−28.70% to 230.38%]	76.48% (60.36%) [−41.82% to 194.79%]	4.74 (0.71) [3.34 to 6.14] PPD^b^	0.69 (0.35) [0.002 to 1.38] PPD^d^	8.67 (7.82) [−6.66 to 24.01] PPD	3.33 (3.35) [−3.24 to 9.89] PPD	14.56 (2.36) [9.94 to 19.18] PPD^b^	155.82 (169.91) [−177.19 to 488.83] PPD
Hispanic vs White difference (SE) [95% CI]	26.81% (4.88%) [17.24% to 36.39%]^b^	29.84% (4.19%) [21.62% to 38.05%]^b^	37.49% (27.07%) [−15.55% to 90.54%]	4.48% (21.96%) [−38.57% to 47.53%]	8.07 (0.87) [6.27 to 9.87] PPD^b^	4.24 (0.47) [3.33 to 5.16] PPD^b^	2.84 (4.13) [−5.25 to 10.93] PPD	−1.34 (2.19) [−5.64 to 2.96] PPD	12.34 (3.48) [5.51 to 19.16] PPD^b^	21.52 (20.22) [−18.12 to 61.16] PPD
Other vs White difference (SE) [95% CI]	−11.17% (4.52%) [−20.04% to −2.31%]^d^	−28.22% (3.20%) [−34.50% to −21.95%]^b^	18.92% (29.21%) [−38.33% to 76.16%]	−0.50% (18.94%) [−37.61% to 36.61%]	2.69 (1.14) [0.45 to 4.93] PPD^d^	−1.91 (0.42) [−2.73 to −1.10] PPD^b^	−5.80 (3.73) [−13.11 to 1.51] PPD	−3.71 (1.75) [−7.14 to −0.29] PPD^d^	5.35 (3.83) [−2.17 to 12.86] PPD	−2.48 (12.13) [−26.25 to 21.30] PPD
Income										
≤50% FPL, low	0.04	0.02	0.02	0.01	33.07	15.07	26.84	12.73	53.75	47.01
>50%-100% FPL, moderate	0.04	0.02	0.02	0.01	31.94	13.45	25.40	11.65	51.62	48.69
>100% FPL, high	0.03	0.02	0.02	0.01	32.59	13.61	23.31	11.12	57.99	38.09
Mod to low difference (SE) [95% CI]	−2.18% (2.55%) [−7.18% to 2.82%]	−6.37% (2.08%) [−10.44% to −2.30%]^c^	−14.81% (5.86%) [−26.28% to −3.32%]^d^	−15.42% (4.58%) [−24.40% to −6.44%^c^]	−1.13 (0.60) [−2.31 to 0.04] PPD	−1.63 (0.25) [−2.12 to −1.14] PPD^b^	−1.44 (1.39) [−4.16 to 1.29] PPD	−1.08 (0.55) [−2.15 to −0.01] PPD^d^	−2.12 (1.88) [−5.80 to 1.56] PPD	1.68 (4.58) [−7.29 to 10.66] PPD
High to low difference (SE) [95% CI]	−5.93% (3.47%) [−12.72% to 0.89%]	−16.72% (2.45%) [−21.58% to −11.86%]^b^	−33.75% (7.61%) [−48.66% to −18.83%]^b^	−31.67% (5.19%) [−41.86% to 21.49%]^b^	−0.48 (0.81) [−2.06 to 1.09] PPD	−1.46 (0.31) [−2.07 to −0.84] PPD^b^	−3.52 (2.07) [−7.58 to 0.53] PPD	−1.61 (0.74) [−3.06 to −0.16] PPD^d^	4.24 (2.80) [−1.25 to 9.74] PPD	−8.92 (6.04) [−20.76 to 2.92] PPD
Education										
Less than high school	0.04	0.02	0.02	0.01	35.91	15.54	24.37	13.36	43.94	36.18
High school or more	0.04	0.02	0.02	0.01	32.11	14.48	26.26	12.51	54.06	50.66
Difference (SE) [95% CI]	−3.20% (3.37%) [−9.80% to 3.40%]	−4.58% (3.10%) [−1.49% to 10.65%]	3.90% (14.02%) [−23.57% to 31.38%]	−1.21% (10.30%) [−21.40% to 18.97%]	−3.80 (0.73) [−5.24 to −2.36] PPD^b^	−1.05 (0.38) [−1.80 to −0.31] PPD^c^	1.88 (2.29) [−2.61 to 6.37] PPD	−0.85 (1.14) [−3.07 to 1.38] PPD	−11.87 (3.32) [−18.38 to −5.37] PPD^b^	−8.32 (9.65) [−27.23 to 10.58] PPD
Age, y										
18-25	0.02	0.01	0.01	0.01	28.65	13.21	23.37	12.79	50.08	39.59
26-35	0.03	0.02	0.03	0.01	32.79	15.83	26.78	13.73	54.16	49.71
36-45	0.04	0.02	0.03	0.01	33.24	15.04	28.36	14.11	57.54	53.81
46-55	0.04	0.02	0.02	0.01	34.08	13.51	24.52	10.47	53.53	44.70
56-64	0.04	0.02	0.02	0.01	31.73	11.47	23.13	7.98	43.09	31.96
18-25 y vs 26-35 y difference (SE) [95% CI]	−34.27% (2.75%) [−39.66% to −28.88%]^b^	−34.82% (2.18%) [−39.10% to −30.54%]^b^	−41.54% (8.41%) [−58.01% to −25.06%]^b^	−25.02% (8.61%) [−41.89% to −8.15%]^c^	−4.15 (0.89) [−5.90 to −2.40] PPD^b^	−2.62 (0.36) [−3.31 to −1.92] PPD^b^	−3.41 (2.58) [−8.46 to 1.65] PPD	−0.94 (1.18) [−3.26 to 1.38] PPD	−4.08 (2.99) [−9.94 to 1.78] PPD	−10.12 (7.77) [−25.35 to 5.10] PPD
36-45 y vs 26-35 y difference (SE) [95% CI]	22.48% (3.28%) [16.04% to 28.91%]^b^	14.20% (2.74%) [8.83% to 19.56%]^b^	3.57% (8.89%) [−13.85% to 20.99%]	3.62% (7.31%) [−10.70% to 17.94%]	0.45 (0.61) [−0.75 to 1.64] PPD	−0.79 (0.28) [−1.33 to −0.25] PPD^c^	1.58 (1.70) [−1.75 to 4.92] PPD	0.38 (0.80) [−1.18 to 1.94] PPD	3.38 (2.29) [−1.10 to 7.86] PPD	4.10 (5.58) [−6.84 to 15.03] PPD
46-55 y vs 26-35 y difference (SE) [95% CI]	30.60% (3.95%) [22.87% to 38.34%]^b^	11.38% (3.16%) [5.19% to 17.58%]^b^	−11.71% (8.99%) [−29.32% to 5.90%]	−19.22% (6.67%) [−32.30% to −6.14%]^c^	1.29 (0.71) [−0.10 to 2.67] PPD	−2.32 (0.31) [−2.92 to −1.71] PPD^b^	−2.26 (1.77) [−5.73 to 1.21] PPD	−3.25 (0.81) [−4.85 to −1.67] PPD^b^	−0.64 (2.53) [−5.60 to 4.33] PPD	−5.01 (5.79) [−16.35 to 6.33] PPD
56-64 y vs 26-35 y difference (SE) [95% CI]	8.08% (4.46%) [−0.66% to 16.81%]	−9.39% (3.42%) [−16.10% to −2.67%]^c^	−31.31% (8.02%) [−47.03% to −15.59%]^b^	−42.14% (5.32%) [−52.57% to −31.71%]^b^	−1.07 (0.95) [−2.92 to 0.79] PPD	−4.36 (0.37) [−5.09 to −3.63] PPD^b^	−3.65 (2.12) [−7.80 to 0.50] PPD	−5.75 (0.81) [−7.34 to −4.17] PPD^b^	−11.07 (2.47) [−15.92 to −6.22] PPD^b^	−17.75 (5.49) [−28.51 to −6.99] PPD^c^
Comorbidities										
Any chronic condition	0.06	0.03	0.04	0.02	34.15	15.75	29.09	14.21	56.58	51.92
No chronic condition	0.02	0.01	0.01	0.01	30.17	12.35	20.71	9.06	48.28^b^	36.41
Difference (SE) [95% CI]	178.93% (6.85%) [165.50% to 192.35%]^b^	176.68% (6.23%) [164.42% to 188.83%]^b^	285.43% (28.22%) [230.13% to 340.74%]^b^	273.68% (19.47%) [235.53% to 311.84%]^b^	3.98 (0.51) [2.99 to 4.97] PPD^b^	3.39 (0.21) [3.00 to 3.81] PPD^b^	8.38 (1.27) [5.89 to 10.88] PPD^b^	5.16 (0.51) [4.15 to 6.16] PPD^b^	8.30 (1.65) [5.06 to 11.54] PPD^b^	15.51 (3.90) [7.87 to 23.16] PPD^b^
No. (person-months)	1 709 196	4 415 423	280 800	725 400	188 925	475 227	25 394	63 422	1 709 196	280 800

Abbreviations: FPL, federal poverty level; PHE, public health emergency.

^a^ Estimates of the change in telehealth visits are from a set of difference-in-difference models where each characteristic is interacted with a post-PHE indicator while controlling for all other characteristics and clustering at the census block group level. Models do include missing data coded as shown in Table 1 but these estimates are not shown. Standard errors on the percent difference are calculated by the delta method.

^b^ *P* < .001.

^c^ *P* < .01.

^d^ *P* < .05.

^e^ Other race non-Hispanic includes American Indian, Asian, and Pacific Islander.

### Chronic Disease Subgroups

Restricting analyses to the CMD, CPD, and SUD subgroups (eTable 1, eTable 2, eTable 3, and eTable 4 in Supplement 1) minimally impacted results with 2 exceptions. First, access to HSI was not associated with telehealth use in the SUD subgroup. Second, in the SUD subgroup, there were no significant differences in telehealth uptake between Hispanic and non-Hispanic White individuals, possibly reflecting sample size (352 participants) (eTable 1 in Supplement 1).

## Discussion

In this cohort study of Wisconsin Medicaid beneficiaries, telehealth expansion played an important role in maintaining access to primary care during the PHE. At its peak, telehealth represented half of all primary care visits and continued to constitute roughly 8% of visits nearly 2 years into the PHE. Unfortunately, expanded telehealth services are not yet guaranteed in the US.^40^ Ensuring continued access to telehealth benefits will require state and federal policy changes.^4,38,40^

Yet, telehealth did not buoy access for all groups equally. In the case of age, sex, and geography, greater telehealth uptake was observed for those receiving more care pre-PHE, specifically, those who were female, middle-aged, and urban. Increased telehealth use among middle-aged^21,41^ and urban residents^22,42^ parallels existing literature. Although findings suggest that telehealth will not equalize primary care utilization, emphasizing telehealth for higher-utilizing groups could still create bandwidth for clinics to better engage lower-utilizing patient populations. Mechanisms underlying telehealth responsiveness are unclear, and the degree to which these differences reflect preferences vs disparities in access to, knowledge of, or agency to utilize telehealth services cannot be determined from findings. Higher utilization could reflect stronger knowledge of the health care system or trust in practitioners empowering these groups to trial telehealth. Digital literacy is unlikely to fully explain differences in telehealth utilization given lower uptake among both younger and older age groups.

Importantly, we observed greater telehealth uptake among individuals with less education and lower income, boosting the pre-PHE visit advantage for the lowest income group and reducing the pre-PHE visit deficit for the lowest education group. However, research has demonstrated tendency toward audio-only visits in these populations,^21,24,41^ and audio-only visits may not offer sufficient quality to support health. Notably, utilization differences by income and education were far smaller than those by geography. Although SES is an important determinant of health,^43^ our findings call for health systems and policymakers to consider the distribution of patients and services by geography to advance health equity.

The association of race and ethnicity with telehealth was more nuanced. Telehealth increased less for non-Hispanic Black individuals than non-Hispanic White individuals but constituted a larger visit share and provided a greater offset for non-Hispanic Black individuals reflecting their lower pre-PHE visit rate. Thus, telehealth helped equalize utilization but did not close this gap. More consistently, Hispanic individuals exhibited greater telehealth uptake across all measures, identifying telehealth as a means of expanding access for this population. Yet, telehealth uptake was the same or lower among non-Hispanic individuals from other races than non-Hispanic White individuals. These findings elucidate conflicting literature demonstrating both higher^41,44^ and lower^24,45^ telehealth utilization in racial and ethnic minority groups relative to White individuals. This inconsistency may reflect variation in telehealth uptake by race or ethnicity or moderation by other factors. For example, in this study, increased telehealth uptake among Hispanic and non-Hispanic Black individuals occurred only among individuals with HSI. These findings inspire important questions about the mechanisms driving these differences: if patients are being triaged into telehealth according to race or ethnicity, telehealth could widen health disparities. Alternatively, if patients from racial and ethnic minority groups are preferentially selecting telehealth to avoid racist encounters, telehealth could reduce disparities given adverse effects of racism on health^46^ (assuming sufficient telehealth quality).^47^ Regarding quality, research has demonstrated lower interest and likelihood of completing video vs phone-only visits among Black, Hispanic or Latino, and Asian individuals relative to White individuals.^19,21,22,24,25,41,45,48^ Again, the effectiveness of phone-only visits is unclear. Regardless, our findings suggest that telehealth expansion is unlikely to fully close primary care utilization gaps by race or ethnicity.

Access to HSI emerged as a key factor related to telehealth use. These findings align with prior literature suggesting that HSI expansion would increase primary care receipt.^33,49,50^ However, differences in telehealth use by sex, geography, age, and income persisted among those with HSI, suggesting that HSI expansion would not close utilization gaps for these groups. Adding complexity, exploratory analyses identified 20% of post-PHE visits as phone-only, inspiring mechanistic questions about how HSI promotes telehealth.

Regarding health status, individuals with chronic disease completed more pre-PHE in-person care and post-PHE telehealth. Telehealth offset over half of their in-person visit decline, highlighting the large residual post-PHE health care deficit for beneficiaries with chronic disease. Findings mirror literature documenting expanded telehealth chronic disease management post-PHE without a net visit increase providing reassurance that telehealth will not induce unnecessary utilization.^51,52,53^ In tandem, HSI expansion is unlikely to promote preventive care among healthy younger adults.

Utilization varied little by disease subgroup. Notably, beneficiaries with SUDs demonstrated high telehealth uptake regardless of HSI access, matching literature showing increased SUD telehealth treatment during the PHE.^54,55,56^ Findings may reflect increased reliance on cellular data in this group due to unstable housing, precluding home-based internet usage. Findings may justify attention to telephone interventions for SUD treatment engagement.

### Limitations

This study had limitations. We required continuous enrollment, so findings may not represent beneficiaries with greater enrollment discontinuity. We used zip codes from March 2020; beneficiaries who subsequently changed zip codes may have been misclassified by internet speed. We did not examine utilization by preferred language, or cluster by practitioner or facility. These factors may influence telehealth use.^57^ Additionally, our findings cannot speak to telehealth visit quality or impact on health outcomes.

## Conclusions

In this study of Wisconsin Medicaid beneficiaries, we found differences in primary care telehealth uptake by age, race, sex, geography, income, and education. In the cases of age, sex, and geography, variation in telehealth uptake paralleled preexisting utilization trends suggesting that telehealth expansion is unlikely to close utilization gaps. In contrast, telehealth may offer a minor advantage in closing utilization gaps by race and education and may strengthen the utilization advantage among individuals with the lowest incomes. HSI promoted telehealth utilization, but we found no evidence that HSI expansion would close utilization disparities. Although telehealth expansion has been touted as a low threshold policy intervention to expand access to care, leveraging telehealth to improve access for underserved populations will require more nuanced attention to the specific mechanisms linking telehealth and health care utilization to avoid inadvertently deepening disparities for select populations.

## Notes

Supplement 1. **eMethods 1. **Identifying Primary Care Visits **eMethods 2. **Identifying Telehealth Visits **eMethods 3.** Diagnosis Codes **eTable 1. **Sociodemographic and Clinical Characteristics of 3 Nonmutually Exclusive Subgroups Defined by Clinical Diagnoses **eTable 2. **Changes in the Use of Telehealth by Access to High-Speed Internet and by Sociodemographic Characteristic Among Beneficiaries With Chronic Medical Disease **eTable 3. **Changes in the Use of Telehealth by Access to High-Speed Internet and by Sociodemographic Characteristic Among Beneficiaries With Chronic Psychiatric Disease **eTable 4. **Changes in the Use of Telehealth by Access to High-Speed Internet and by Sociodemographic Characteristic Among Beneficiaries With Substance Use Disorders

Supplement 2.
Data Sharing Statement
