# Supplementary material for: Telehealth Expansion, Internet Speed, and Primary Care Access Before and During COVID-19
Source: JAMA Netw Open. 2024 Jan 5;7(1):e2347686. doi: 10.1001/jamanetworkopen.2023.47686 (PMC10770767; doi:10.1001/jamanetworkopen.2023.47686)
Supplement: Supplement 1. — eMethods 1. Identifying Primary Care Visits eMethods 2. Identifying Telehealth Visits eMethods 3. Diagnosis Codes eTable 1. Sociodemographic and Clinical Characteristics of 3 Nonmutually Exclusive Subgroups Defined by Clinical Diagnoses eTable 2. Changes in the Use of Telehealth by Access to High-Speed Internet and by Sociodemographic Characteristic Among Beneficiaries With Chronic Medical Disease eTable 3. Changes in the Use of Telehealth by Access to High-Speed Internet and by Sociodemographic Characteristic Among Beneficiaries With Chronic Psychiatric Disease eTable 4. Changes in the Use of Telehealth by Access to High-Speed Internet and by Sociodemographic Characteristic Among Beneficiaries With Substance Use Disorders [file jamanetwopen-e2347686-s001.pdf]

Supplemental Online Content

Tilhou AS, Jain A, DeLeire T. Telehealth expansion, internet speed, and primary care access before and during COVID-19. *JAMA Netw Open*. 2023;6(12):e2347686. doi:10.1001/jamanetworkopen.2023.47686

eMethods 1. Identifying Primary Care Visits
eMethods 2. Identifying Telehealth Visits
eMethods 3. Diagnosis Codes
eTable 1. Sociodemographic and Clinical Characteristics of 3 Nonmutually Exclusive Subgroups Defined by Clinical Diagnoses
eTable 2. Changes in the Use of Telehealth by Access to High-Speed Internet and by Sociodemographic Characteristic Among Beneficiaries With Chronic Medical Disease
eTable 3. Changes in the Use of Telehealth by Access to High-Speed Internet and by Sociodemographic Characteristic Among Beneficiaries With Chronic Psychiatric Disease
eTable 4. Changes in the Use of Telehealth by Access to High-Speed Internet and by Sociodemographic Characteristic Among Beneficiaries With Substance Use Disorders

This supplemental material has been provided by the authors to give readers additional information about their work.

© 2023 Tilhou AS et al. *JAMA Network Open.*

eMethods 1. Identifying Primary Care Visits

To identify primary care visits, as opposed to other kinds of outpatient visits, we used the following provider specialty codes from Wisconsin Medicaid program documentation:
320 - geriatrics
316 – family practice
271 – general practice
318 – general practice
92 – nurse practitioner family practice

In addition, a number of provider specialty codes do not clearly indicate a specialty, or are a place of service without an associated specialty.
322 – internal medicine
100 – "Physician Assistant"
125 – "advanced practice nurse prescriber"
126 – "qualified treatment trainee"
184 - "hospital affiliated clinic"
185 - "free standing clinic"
300 - "free standing"
301 - "hospital affiliated"

To assess whether these codes represented primary care services, we cross-tabulated these codes with the rendering provider taxonomy. Based on cross-tabulations, we included visits that were likely primary care because either a) the visit occurs in a typical primary care setting, e.g. FQHC, CHC, Rural Health Center, etc., and neither the taxonomy nor the provider code indicate a non-primary care specialty, or b) the provider taxonomy indicated general practice (internal medicine or family medicine) along with a specialty often embedded in primary care practices (e.g., sleep medicine, sports medicine, obesity, addiction). In contrast, if the codes indicated internal medicine and a specialty such as pulmonology or cardiology then those visits were excluded. We also excluded visits for women's health, family planning, and pediatrics

Finally, the following provider codes did not appear during our study period and were excluded: 80-FQHC, 81 -CHC, 83 – family planning, 93 – other NP, 95 – NP/nurse midwife.

© 2023 Tilhou AS et al. *JAMA Network Open.*

eMethods 2. Identifying Telehealth Visits

We identified the use of telehealth, as opposed to in-person services, based on the presence of a procedure code, or the presence of either a place of service code or modifier indicating telehealth.
Modifier: GT or 95
Place of service indicator: POS 02
Procedure codes:
E-visits: 98970, 98971, 89872, 99421, 99422, 99423
Telephone E&M Services: 99441, 99442, 99443

eMethods 3. Diagnosis Codes

Chronic Medical Diseases
Asthma: J4520, J4521, J4522, J4530, J4531, J4532, J4540, J4541, J4542, J4550, J4551, J4552, J45901, J45902, J45909, J45990, J45991, J45998
Chronic kidney disease: I120-I132, N181-N189, F880, Z4901-4902, Z4921-4932
Chronic obstructive pulmonary disease: J410, J411, J418, J42, J430, J431, J432, J439, J440, J441, J449, J470, J471, J479
Coronary artery disease: I200 I201 I208, I209 I2101 I2102 I2109 I2111 I2119 I2121 I2129 I213 I214 I219 I21A1, I21A9, I220, I221, I222, I228, I229, I240, I248, I249, I2510, I25110, I25111, I25118, I25119, I252, I255, I256, I25700, I25701, I25708, I25709, I25710, I25711, I25718, I25719, I25720, I25721, I25728, I25729, I25730, I25731, I25738, I25739, I25750, I25751, I25758, I25759 I25760 I25761 I25768 I25769 I25790 I25791 I25798 I25799 I25810 I25811 I25812 I2582 I2583 I2584 I2589 I259 Z951 Z955 Z9861
Diabetes: exhaustive list of ICD10 diabetes codes from E0800 to E139
Hypertension: I10, I119, I150, I151, I152, I158, I159, I160, I161, I169, I1674
Thyroid disease: B6731 and E001 – E890
Heart failure: I50-I50.9
Osteoarthritis: M15.0, M15.1, M15.2, M15.3, M15.8, M16.10, M16.7, M16.9, M17.10, M17.5, M17.9, M18.9, M19.019, M19029, M19.039, M19.049, M19.079, M19.219, M19.229, M19.239, M19.249, M19.279, M19.90, M19.91, M19.93

Chronic Psychiatric Diseases
We used ICD-10 codes F200-F99 except F550-F59 (anabolic steroid abuse) and F430 and F4311 (Aaute stress reaction)

Substance Use Disorders
We used ICD-10 codes for substance use in the following families: alcohol (F10), opioids (F11), cannabis (F12), sedatives (F13), stimulants (F14 and F15), hallucinogens (F16), inhalants (F18) and other psychoactive substances (F19). We excluded nicotine (F17) and miscellaneous SUDs (F550-F558).

© 2023 Tilhou AS et al. *JAMA Network Open.*

eTable 1. Sociodemographic and Clinical Characteristics of 3 Nonmutually Exclusive Subgroups Defined by Clinical Diagnoses

	CMD		CPD		SUD	
	N = 37,671		N = 47,017		N = 16,837	
Characteristics	N	Percent	N	Percent	N	Percent
Female	23,429	62.2%	32,936	70.1%	8,502	50.5%
Male	14,242	37.8%	14,081	29.9%	8,335	49.5%
Black non-Hispanic	7,936	21.1%	5,373	11.4%	2,197	13.0%
Hispanic	3,210	8.5%	3,474	7.4%	1,083	6.4%
Other Race non-Hispanic	2,563	6.8%	2,612	5.6%	1,215	7.2%
White non-Hispanic	22,624	60.1%	34,371	73.1%	11,912	70.7%
Missing Race/Ethnicity	1,338	3.6%	1,187	2.5%	430	2.6%
Income [0, 50]% FPL	24,183	64.2%	31,466	66.9%	13,355	79.3%
Income (50, 100]% FPL	11,513	30.6%	13,024	27.7%	2,979	17.7%
Income > 100% FPL	1,974	5.2%	2,526	5.4%	503	3.0%
Missing Income	< 10	0.0%	< 10	0.0%	0	0.0%
Rural County	8,224	21.8%	11,348	24.1%	3,738	22.2%
Urban County	24,420	64.8%	29,765	63.3%	10,809	64.2%
Missing County	5,027	13.3%	5,904	12.6%	2,290	13.6%
Low Speed Internet	5,043	13.4%	6,171	13.1%	1,966	11.7%
High Speed Internet	31,267	83.0%	39,041	83.0%	14,122	83.9%
Missing Census Block Group	1,361	3.6%	1,805	3.8%	749	4.4%
Less than High School	6,840	18.2%	6,742	14.3%	3,215	19.1%
High School or More	21,900	58.1%	29,920	63.6%	10,210	60.6%
Missing Education	8,931	23.7%	10,355	22.0%	3,412	20.3%
Age 18-25	2,165	5.7%	5,420	11.5%	1,331	7.9%
Age 26-35	7,494	19.9%	15,890	33.8%	6,181	36.7%
Age 36-45	11,144	29.6%	14,409	30.6%	5,252	31.2%
Age 46-55	10,787	28.6%	8,245	17.5%	3,026	18.0%
Age 56-64	6,081	16.1%	3,053	6.5%	1,047	6.2%
Any Chronic Condition	37,671	100.0%	47,017	100.0%	16,837	100.0%
No Chronic Condition	0	0.0%	0	0.0%	0	0.0%
Chronic Medical Disease	37,671	100.0%	16,864	35.9%	5,316	31.6%
Chronic Psychiatric Disease	16,864	44.8%	47,017	100.0%	10,565	62.7%
Substance Use Disorder	5,316	14.1%	10,565	22.5%	16,837	100.0%

Abbreviations: CMD, chronic medical disease; CPD, chronic psychiatric disease; SUD, substance use disorder; FPL, federal poverty level
Other Race non-Hispanic includes American Indian, Asian, and Pacific Islander.

© 2023 Tilhou AS et al. *JAMA Network Open.*

eTable 2. Changes in the Use of Telehealth by Access to High-Speed Internet and by Sociodemographic Characteristic Among Beneficiaries With Chronic Medical Disease

© 2023 Tilhou AS et al. *JAMA Network Open.*

N=37671	Change in Monthly TH Visits June 2019 to May 2020	Change in Monthly TH Visits June 2019 to December 2021	Change in TH Share June 2019 to May 2020	Change in TH Share June 2019 to December 2021	TH Offset June 2019 to May 2020
County					
Rural	0.04	0.02	25.26	11.24	45.24
Urban	0.07	0.03	36.56	15.76	59.21
Difference (SE) [95%CI]	80.08***	68.80***	11.29***	4.52***	13.97***
	(9.01)	(7.11)	(1.05)	(0.45)	(3.01)
	(62.42 to 97.75)	(54.87 to 82.73)	(9.23 to 13.35)	(3.64 to 5.39)	(8.07 to 19.87)
Sex					
Female	0.06	0.03	34.83	15.62	60.83
Male	0.05	0.02	33.76	13.34	50.73
Difference (SE) [95%CI]	18.65***	34.34***	1.07	2.29***	10.10***
	(4.03)	(4.53)	(0.82)	(0.41)	(2.38)
	(10.74 to 26.56)	(25.47 to 43.21)	(-0.54 to 2.67)	(1.48 to 3.09)	(5.44 to 14.76)
Race and Ethnicity					
Black non-Hispanic	0.07	0.03	39.20	16.73	67.33
Hispanic	0.09	0.04	43.02	19.89	63.97
Other non-Hispanic	0.07	0.03	35.79	12.57	62.52
White non-Hispanic	0.05	0.03	31.25	13.67	51.33
Black vs White difference (SE) [95%CI]	24.75***	15.32**	7.95***	3.07***	16.00***
	(4.98)	(5.09)	(1.06)	(0.53)	(3.14)
	(14.98 to 34.51)	(5.33 to 25.30)	(5.88 to 10.03)	(2.02 to 4.11)	(9.85 to 22.16)
Hispanic vs White difference (SE) [95%CI]	60.85***	66.59***	11.77***	6.23***	12.65**
	(8.65)	(7.80)	(1.48)	(0.73)	(3.76)
	(43.89 to 77.81)	(51.32 to 81.87)	(8.86 to 14.68)	(4.80 to 7.65)	(5.28 to 20.01)
Other vs White difference (SE) [95%CI]	28.29	2.46	4.54**	-1.1	11.2
	(9.53)	(6.75)	(1.70)	(0.64)	(5.77)
	(9.62 to 46.96)	(-10.76 to 15.68)	(1.21 to 7.87)	(-2.36 to 0.16)	(-0.11 to 22.50)
Income					
≤50% FPL	0.06	0.03	34.68	15.26	56.06
>50-100% FPL	0.06	0.03	33.25	13.70	56.24
>100% FPL	0.06	0.03	36.92	15.32	68.09
Mod vs Low difference (SE) [95%CI]	-5.61	-8.08**	-1.43	-1.56***	0.19
	(3.36)	(2.85)	(0.94)	(0.40)	(2.69)
	(-12.19 to 0.98)	(-13.66 to -2.50)	(-3.27 to 0.41)	(-2.34 to -0.79)	(-5.10 to 5.47)
High vs Low difference (SE) [95%CI]	-0.56	-9.51*	2.23	0.06	12.03*
	5.64	(4.22)	(1.45)	(0.61)	(5.30)
	(-11.61 to 10.50)	(-17.79 to -1.23)	(-0.60 to 5.07)	(-1.15 to 1.26)	(1.65 to 22.42)
Education					
Less than high school	0.07	0.03	38.95	16.71	47.79
High school or more	0.06	0.03	33.71	14.86	57.27
Difference (SE) [95%CI]	-14.95***	-5.53	-5.24***	-1.85**	-11.41**
	(3.83)	(3.74)	(1.09)	(0.54)	(4.02)
	(-22.45 to -7.45)	(-12.86 to 1.80)	(-7.38 to -3.11)	(-2.90 to -0.79)	(-19.29 to -3.54)
Age, y					
18-25	0.04	0.02	31.72	15.56	58.35
26-35	0.06	0.03	34.88	16.91	60.04
36-45	0.07	0.03	34.77	15.87	60.83
46-55	0.06	0.03	34.53	13.68	56.00
56-64	0.05	0.02	33.91	12.06	47.77
(18-25) vs (26-35) difference (SE) [95%CI]	-26.43***	-26.31***	-3.16	-1.35	-1.69
	(5.99)	(5.88)	(1.88)	(0.99)	(6.91)
	(-38.17 to -14.69)	(-37.84 to -14.78)	(-6.85 to 0.53)	(-3.28 to 0.58)	(-15.23 to 11.85)
(36-45) vs (26-35) difference (SE) [95%CI]	9.49	1.70	-0.11	-1.04	0.79
	(4.99)	(4.45)	(1.10)	(0.54)	(3.80)
	(-0.29 to 19.27)	(-7.02 to 10.41)	(-2.26 to 2.05)	(-2.09 to 0.01)	(-6.66 to 8.23)
(46-55) vs (26-35) difference (SE) [95%CI]	1.44	-14.03***	-0.35	-3.23***	-4.04
	(4.69)	(3.90)	(1.10)	(0.52)	(3.59)
	(-7.75 to 10.62)	(-21.68 to -6.38)	(-2.50 to 1.81)	(-4.25 to -2.22)	(-11.09 to 3.01)
(56-64) vs (26-35) difference (SE) [95%CI]	-14.10**	-29.30***	-0.97	-4.85***	-12.28**
	(4.66)	(3.68)	(1.34)	(0.58)	(3.76)
	(-23.23 to -4.98)	(-36.52 to -22.08)	(-3.59 to 1.66)	(-5.98 to -3.72)	(-19.64 to -4.91)
Internet					
High speed	0.06	0.03	35.36	15.11	57.81
Low speed	0.04	0.02	27.36	12.41	49.88
Difference (SE) [95%CI]	62.23***	54.21***	8.00***	2.70***	7.93*
	(9.94)	(8.20)	(1.28)	(0.58)	(3.69)
	(42.75 to 81.71)	(38.13 to 70.29)	(5.50 to 10.50)	(1.56 to 3.83)	(0.69 to 15.16)

© 2023 Tilhou AS et al. *JAMA Network Open.*

*p<0.05, **p<0.01
Notes: Pre-PHE visits are unadjusted. Estimates of the change in telehealth visits are from a set of difference-in-difference models where each characteristic is interacted with a post-PHE indicator controlling for all other characteristics. Standard errors on the percent difference are calculated by the delta method. Other Race non-Hispanic includes American Indian, Asian, and Pacific Islander.
Abbreviations: TH, telehealth; FPL, federal poverty level; B, Black; W, White, H, Hispanic; O, Other race

© 2023 Tilhou AS et al. *JAMA Network Open.*

eTable 3. Changes in the Use of Telehealth by Access to High-Speed Internet and by Sociodemographic Characteristic Among Beneficiaries With Chronic Psychiatric Disease

© 2023 Tilhou AS et al. *JAMA Network Open.*

n=47017	Change in TH Visits<br>June 2019 to May 2020	Change in TH Visits<br>June 2019 to December 2021	Change in TH Share<br>June 2019 to May 2020	Change in TH Share<br>June 2019 to December 2021	TH Offset<br>June 2019 to May 2020
County					
Rural	0.04	0.02	27.43	13.70	47.98
Urban	0.06	0.03	35.76	17.63	56.52
Difference (SE) [95%CI]	51.52***	54.43***	8.34***	3.94***	8.53**
	(6.44)	(5.55)	(0.91)	(0.45)	(2.67)
	(38.90 to 64.14)	(43.55 to 65.30)	(6.55 to 10.12)	(3.07 to 4.81)	(3.29 to 13.78)
Sex					
Female	0.06	0.03	34.19	17.19	57.91
Male	0.05	0.03	33.88	15.66	49.68
Difference (SE) [95%CI]	13.66***	23.93***	0.31	1.53***	8.23***
	(3.90)	(4.09)	(0.83)	(0.44)	(2.29)
	(6.02 to 21.30)	(15.91 to 31.95)	(-1.33 to 1.94)	(0.67 to 2.39)	(3.75 to 12.72)
Race and Ethnicity					
Black non-Hispanic	0.07	0.03	38.34	18.10	68.01
Hispanic	0.07	0.04	39.96	19.99	59.97
Other non-Hispanic	0.06	0.03	33.49	14.79	52.98
White non-Hispanic	0.06	0.03	32.85	16.38	53.30
Black vs White difference (SE) [95%CI]	21.51***	10.52*	5.49***	1.73**	14.71***
	(5.67)	(4.81)	(1.30)	(0.71)	(3.78)
	(10.40 to 32.63)	(1.10 to 19.94)	(2.95 to 8.03)	(0.34 to 3.11)	(7.30 to 22.12)
Hispanic vs White difference (SE) [95%CI]	33.14***	32.45***	7.12***	3.61***	6.67
	(6.88)	(6.15)	(1.39)	(0.74)	(4.27)
	(19.66 to 46.62)	(20.40 to 44.50)	(4.40 to 9.83)	(2.16 to 5.06)	(-1.69 to 15.04)
Other vs White difference (SE) [95%CI]	2.57	-7.48	0.64	-1.59*	-0.32
	(7.37)	(6.28)	(1.73)	(0.78)	(4.76)
	(-11.87 to 17.02)	(-19.78 to 4.82)	(-2.75 to 4.03)	(-3.12 to -0.06)	(-9.65 to 9.00)
Income					
≤50% FPL	0.06	0.03	34.48	17.26	54.55
>50-100% FPL	0.05	0.03	32.87	15.76	55.35
>100% FPL	0.05	0.03	34.94	15.99	63.43
Mod vs Low difference (SE) [95%CI]	-9.16**	-9.97***	-1.61	-1.50***	0.8
	(3.07)	(2.64)	(0.84)	(0.38)	(2.59)
	(-15.18 to -3.14)	(-15.15 to -4.78)	(-3.26 to 0.04)	(-2.25 to -0.76)	(-4.28 to 5.88)
High vs Low difference (SE) [95%CI]	-8.15	-19.05***	0.46	-1.27*	8.88
	(4.49)	(3.16)	(1.25)	(0.53)	(4.56)
	(-16.95 to 0.65)	(-25.25 to -12.85)	(-1.99 to 2.91)	(-2.30 to -0.23)	(-0.05 to 17.81)
Education					
Less than high school	0.07	0.03	36.61	17.92	47.20
High school or more	0.06	0.03	33.20	16.56	56.61
Difference (SE) [95%CI]	-12.31**	-5.14	-3.41**	-1.36*	-7.22
	(3.86)	(3.97)	(1.08)	(0.64)	(4.01)
	(-19.88 to -4.74)	(-12.92 to 2.64)	(-5.53 to -1.29)	(-2.62 to -0.11)	(-15.08 to 0.64)
Age, y					
18-25	0.04	0.02	30.62	15.79	50.59
26-35	0.06	0.03	33.68	17.94	54.65
36-45	0.06	0.03	35.06	17.57	60.14
46-55	0.07	0.03	35.40	15.19	56.08
56-64	0.05	0.03	32.05	13.13	42.15
(18-25) vs (26-35) difference (SE) [95%CI]	-34.08***	-29.97***	-3.06*	-2.14***	-4.05
	(3.80)	(3.53)	(1.35)	(0.61)	(4.39)
	(-41.52 to -26.63)	(-36.88 to -23.05)	(-5.70 to -0.42)	(-3.34 to -0.95)	(-12.66 to 4.55)
(36-45) vs (26-35) difference (SE) [95%CI]	15.76***	7.79*	1.38	-0.37	5.49
	(4.20)	(3.64)	(0.86)	(0.43)	(2.93)
	(7.53 to 23.99)	(0.66 to 14.91)	(-0.30 to 3.06)	(-1.22 to 0.49)	(-0.25 to 11.22)
(46-55) vs (26-35) difference (SE) [95%CI]	17.11**	-3.11	1.72	-2.74***	1.43
	(4.99)	(3.92)	(1.01)	(0.48)	(3.22)
	(7.34 to 26.88)	(-10.80 to 4.57)	(-0.26 to 3.70)	(-3.69 to -1.79)	(-4.88 to 7.74)
(56-64) vs (26-35) difference (SE) [95%CI]	-9.68	-18.08***	-1.63	-4.80***	-12.50**
	(5.59)	(4.85)	(1.51)	(0.64)	(3.75)
	(-20.64 to 1.28)	(-27.58 to -8.59)	(-4.59 to 1.33)	(-6.07 to -3.54)	(-19.86 to -5.14)
Internet					
High speed	0.06	0.03	34.53	16.88	55.50
Low speed	0.05	0.03	30.95	15.85	54.71
Difference (SE) [95%CI]	29.62***	27.66***	3.58**	1.04	0.79
	(6.71)	(5.80)	(1.18)	(0.61)	(3.45)
	(16.47 to 42.78)	(16.30 to 39.03)	(1.27 to 5.90)	(-0.17 to 2.24)	(-5.97 to 7.56)

© 2023 Tilhou AS et al. *JAMA Network Open.*

*p<0.05, **p<0.01
Notes: Pre-PHE visits are unadjusted. Estimates of the change in telehealth visits are from a set of difference-in-difference models where each characteristic is interacted with a post-PHE indicator controlling for all other characteristics. Standard errors on the percent difference are calculated by the delta method. Other Race non-Hispanic includes American Indian, Asian, and Pacific Islander.
Abbreviations: TH, telehealth; FPL, federal poverty level; B, Black; W, White, H, Hispanic; O, Other race

eTable 4. Changes in the Use of Telehealth by Access to High-Speed Internet and by Sociodemographic Characteristic Among Beneficiaries With Substance Use Disorders

© 2023 Tilhou AS et al. *JAMA Network Open.*

	Change in TH Visits		Change in TH Share		TH Offset
n=16837	June 2019 to May 2020	June 2019 to December 2021	June 2019 to May 2020	June 2019 to December 2021	June 2019 to May 2020
County					
Rural	0.05	0.03	27.82	15.87	43.23
Urban	0.06	0.04	31.52	17.29	66.44
Difference (SE) [95%CI]	15.69	19.26*	3.70*	1.42	23.20***
	(9.24)	(7.76)	(1.49)	(0.85)	(6.44)
	(-2.42 to 33.80)	(4.04 to 34.48)	(0.78 to 6.62)	(-0.25 to 3.09)	(10.58 to 35.82)
Sex					
Female	0.07	0.04	32.06	18.23	68.06
Male	0.05	0.03	29.24	15.43	50.98
Difference (SE) [95%CI]	40.20***	49.28***	2.83*	2.80***	17.08**
	(7.12)	(7.28)	(1.14)	(0.67)	(6.45)
	(26.25 to 54.15)	(35.02 to 63.55)	(0.59 to 5.07)	(1.50 to 4.11)	(4.43 to 29.73)
Race and Ethnicity					
Black non-Hispanic	0.05	0.02	35.11	16.11	72.26
Hispanic	0.06	0.03	31.66	17.80	44.55
Other non-Hispanic	0.06	0.03	29.54	12.88	64.21
White non-Hispanic	0.07	0.04	30.56	17.47	60.23
Black vs White difference (SE) [95%CI]	-26.71***	-39.37***	4.55*	-1.36	12.03
	(6.26)	(4.57)	(1.88)	(0.89)	(17.41)
	(-38.97 to -14.45)	(-48.33 to -30.40)	(0.87 to 8.23)	(-3.11 to 0.39)	(-22.09 to 46.15)
Hispanic vs White difference (SE) [95%CI]	-11.93	-13.50	1.10	0.33	-15.68
	(9.61)	(7.64)	(2.60)	(1.32)	(10.09)
	(-30.77 to 6.91)	(-28.47 to 1.47)	(-3.99 to 6.19)	(-2.26 to 2.91)	(-35.47 to 4.10)
Other vs White difference (SE) [95%CI]	-13.53	-28.23**	-1.02*	-4.59***	3.98
	(14.27)	(8.47)	(2.67)	1.27	19.17
	(-41.50 to 14.44)	(-44.82 to -11.64)	(-6.25 to 4.20)	(-7.07 to -2.10)	(-33.59 to 41.55)
Income					
≤50% FPL	0.06	0.03	30.51	16.94	59.46
>50-100% FPL	0.06	0.04	30.79	16.97	60.48
>100% FPL	0.07	0.03	35.70	17.38	63.46
Mod vs Low difference (SE) [95%CI]	5.13	1.98	0.27	0.04	1.02
	(6.91)	(5.95)	(1.51)	(0.78)	(7.12)
	(-8.41 to 18.66)	(-9.69 to 13.64)	(-2.68 to 3.23)	(-1.49 to 1.56)	(-12.93 to 14.97)
High vs Low difference (SE) [95%CI]	10.53	-0.27	5.19	0.45	4.00
	(12.72)	(9.74)	(2.78)	1.39	(11.31)
	(-14.39 to 35.46)	(-19.37 to 18.83)	(-0.26 to 10.64)	(-2.27 to 3.17)	(-18.16 to 26.16)
Education					
Less than high school	0.06	0.03	32.44	16.19	47.27
High school or more	0.06	0.04	30.78	17.30	59.45
Difference (SE) [95%CI]	9.92	29.99**	-1.66	1.10	-23.57
	(8.10)	(8.81)	(1.52)	(0.84)	(19.57)
	(-5.95 to 25.80)	(12.72 to 47.27)	(-4.64 to 1.32)	(-0.55 to 2.75)	(-61.94 to 14.79)
Age, y					
18-25	0.04	0.02	24.96	15.80	63.66
26-35	0.06	0.04	30.18	17.90	54.44
36-45	0.07	0.04	32.28	17.69	69.52
46-55	0.06	0.03	31.94	15.60	67.82
56-64	0.04	0.02	28.76	12.28	33.03
(18-25) vs (26-35) difference (SE) [95%CI]	-39.54***	-36.05***	-5.23*	-2.11	9.22
	(8.28)	(7.45)	(2.53)	(1.36)	(19.56)
	(-55.77 to -23.30)	(-50.66 to 21.45)	(-10.19 to -0.26)	(-4.77 to 0.55)	(-29.11 to 47.55)
(36-45) vs (26-35) difference (SE) [95%CI]	7.70	-2.27	2.09	-0.21	15.08
	(6.90)	(5.65)	(1.34)	(0.77)	(8.66)
	(-5.82 to 21.22)	(-13.35 to 8.80)	(-0.53 to 4.72)	(-1.73 to 1.30)	(-1.90 to 32.06)
(46-55) vs (26-35) difference (SE) [95%CI]	-7.50	-17.63**	1.76	-2.31**	13.39
	(6.98)	(5.54)	(1.64)	0.87	(12.39)
	(-21.17 to 6.17)	(-28.50 to -6.77)	(-1.45 to 4.97)	(-4.01 to -0.60)	(-10.90 to 37.68)
(56-64) vs (26-35) difference (SE) [95%CI]	-35.03***	-39.46***	-1.42	-5.62***	-21.41**
	(8.16)	(7.08)	(2.65)	1.23	(6.22)
	(-51.01 to -19.04)	(-53.33 to -25.59)	(-6.62 to 3.78)	(-8.04 to -3.21)	(-33.60 to -9.22)
Internet					
High speed	0.06	0.03	30.95	16.81	59.91
Low speed	0.06	0.03	29.73	18.18	59.34
Difference (SE) [95%CI]	2.80	0.29	1.22	-1.37	0.57
	(11.44)	(8.54)	(1.95)	(1.32)	(7.74)
	(-19.61 to 25.22)	(-16.46 to 17.03)	(-2.60 to 5.04)	(-.3.96 to 1.21)	(-14.61 to 15.75)

© 2023 Tilhou AS et al. *JAMA Network Open.*

*p<0.05, **p<0.01

Notes: Pre-PHE visits are unadjusted. Estimates of the change in telehealth visits are from a set of difference-in-difference models where each characteristic is interacted with a post-PHE indicator controlling for all other characteristics. Standard errors on the percent difference are calculated by the delta method. Other Race non-Hispanic includes American Indian, Asian, and Pacific Islander.

Abbreviations: TH, telehealth; FPL, federal poverty level; B, Black; W, White, H, Hispanic; O, Other race

© 2023 Tilhou AS et al. *JAMA Network Open.*
