# Supplementary material for: Telehealth Expansion, Internet Speed, and Primary Care Access Before and During COVID-19
Source: JAMA Netw Open. 2024 Jan 5;7(1):e2347686. doi: 10.1001/jamanetworkopen.2023.47686 (PMC10770767; doi:10.1001/jamanetworkopen.2023.47686)
Supplement: Supplement 2. — Data Sharing Statement [file jamanetwopen-e2347686-s002.pdf]

Data Sharing Statement

Tilhou. Telehealth Expansion, Internet Speed, and Primary Care Access Before and During COVID-19. *JAMA Netw Open*. Published December 14, 2023.
doi:10.1001/jamanetworkopen.2023.47686

Data
Data available: No

Additional Information
Explanation for why data not available: The data for this paper are not publicly available. The paper uses linkages of proprietary data from the Wisconsin Department of Health Services housed at the Institute for Research on Poverty at the University of Wisconsin. The construction of and access to these data are governed by data sharing agreements between the University of Wisconsin and the agency that prohibit any redisclosure of the data. Sharing of individual, row-level data is strictly prohibited by each of these existing data use agreements. This prohibition extends to derived datasets based on the raw claims data that aggregate claims, for example, at the person- or person-year level and all data sets to which they are linked at the person-level. Others interested in using the data may inquire through IRP at https://www.irp.wisc.edu/wadc/ and apply for permission.
